# Fabrication of Gold-Coated Ultra-Thin Anodic Porous Alumina Substrates for Augmented SERS

**DOI:** 10.3390/ma9060403

**Published:** 2016-05-24

**Authors:** Chiara Toccafondi, Remo Proietti Zaccaria, Silvia Dante, Marco Salerno

**Affiliations:** 1 Nanophysics Department, Istituto Italiano di Tecnologia, Genova 16163, Italy; chiara.toccafondi@polytechnique.edu (C.T.); silvia.dante@iit.it (S.D.); 2 Laboratoire de Physique des Interfaces et des Couches Minces (LPICM), Centre National de la Recherche Scientifique (CNRS), Ecole Polytechnique, Université Paris Saclay, Palaiseau 91128, France; 3 Nanostructures Department Istituto Italiano, di Tecnologia, Genova 16163, Italy; remo.proietti@iit.it

**Keywords:** SERS, nanostructures, anodic porous alumina, mercaptobenzoic acid, enhancement, surface plasmons, hotspots

## Abstract

Anodic porous alumina (APA) is a nanostructured material used as a template in several nanotechnological applications. We propose the use of APA in ultra-thin form (<100 nm) for augmented surface-enhanced Raman scattering (SERS). Here, the effect of in-depth thinning of the APA nanostructures for possible maximization of SERS was addressed. Anodization was carried out on ultra-thin films of aluminum on glass and/or silicon, followed by pore-opening. Gold (Au) was overcoated and micro-Raman/SERS measurements were carried out on test target analytes. Finite integration technique simulations of the APA-Au substrate were used both for the experimental design and simulations. It was observed that, under optimized conditions of APA and Au thickness, the SERS enhancement is higher than on standard APA-Au substrates based on thin (~100 nm) APA by up to a factor of ~20 for test molecules of mercaptobenzoic acid. The agreement between model and experimental results confirms the current understanding of SERS as being mainly due to the physical origin of plasmon resonances. The reported results represent one step towards micro-technological, integrated, disposable, high-sensitivity SERS chemical sensors and biosensors based on similar substrates.

## 1. Introduction

Anodic porous alumina (APA) is a nanostructured material obtained through controlled anodization of aluminum (Al) substrates [1]. In APA, the oxide layer formed on the metal surface is nanoporous, with a pore diameter tunable in a broad range of approximately 10–300 nm and relatively monodispersed [2]. As-anodized APA is amorphous; however, post-fabrication annealing may be used to convert it to crystalline phase [3]. APA may find important primary applications in both photonics, for example, to make photonic crystals [4] and distributed feedback lasers [5], and in biological and biomedical studies as a substrate with controlled nanoscale roughness for optimized adhesion and the growth of living cells in cultures [6,7]. In the latter field, APA has also been proposed as a substrate for drug delivery, taking advantage of the volume of the nanopores for loading and the subsequent localized elution of bioactive agents (molecules or nanoparticles) [8,9,10]. In an effort to combine both above-mentioned properties, namely the possible optoelectronic functionality of the nanostructure with the ability to sustain living cells on its surface, in our group, we have recently started to assemble dedicated APA-based devices aimed to perform surface-enhanced Raman scattering (SERS) spectroscopy of living cells [11]. To this goal, the APA surface is coated with a thin layer of gold (Au), which adopts the APA nanostructured profile, providing the physical enhancement of the Raman signal thanks to the plasmonic effect. Silver (Ag) is often used as well for SERS [12], also on APA substrates [13,14,15], since it shows even stronger plasmon resonances than Au [16]. Yet Ag is prone to oxidation and does not provide a chemically interesting surface like Au, which is open to thiol chemistry in view of proper bio-functionalization. For these reasons, we selected Au as the APA coating. Similarly to natural lithography made by Au deposition through masks of nano-spheres [17], coating APA with Au is a method based on self-organization of the substrate, allowing large-area SERS surfaces to be obtained by inexpensive means. While for initial SERS experiments we used thick APA, formed from the anodization of thick (~100 µm scale) Al foils [18], we have recently moved on to the anodization of thin Al films (~100 nm) previously coated on technological substrates of either silicon (Si) or optical quality glass wafers, which we called thin APA (tAPA). tAPA should permit the integration of the SERS substrate, comprising the APA surface and the Au coating (APA-Au) with the available technological processing capabilities of the microelectronics industry, allowing for the lithographic patterning of prototype topologies of potential biosensors or bioassay devices. However, the controlled fabrication of Au-coated tAPA substrates (tAPA-Au) is not trivial since the quality of the starting Al film is critical, and the anodization process is short.

In this work, we propose one further step in optimization of our substrates for SERS, namely the use of ultra-thin APA (utAPA, below ~40-nm thickness) as the surface for Au coating. As compared to different optimization work [19], we focus on the thickness of the underlying APA as the primary parameter instead of that of the overcoating Au. Our resulting utAPA-Au substrates should take advantage of the additional coupling of surface plasmons in the Au between the top (edges of the pore mouth walls) and the bottom of the APA pores, which is easily reached by the coated Au when using APA of such a low thickness. Obviously, the fabrication of these utAPA-Au substrates is even more critical from the technical point of view. Additionally, in order to make an optimal design and to confirm the experimental results, the expected effect of these substrates has to be obtained by modeling and simulation with the appropriate software tool. For this reason, here the electrical field enhancement at the SERS hotspots resulting from laser irradiation of a given utAPA-Au nanostructure during the SERS measurements have been calculated by means of a finite integration technique program and compared with the results of measurements performed with a selected test probe molecule for SERS.

## 2. Results and Discussion

### 2.1. utAPA-Au Substrate Preparation

**Figure 1:**
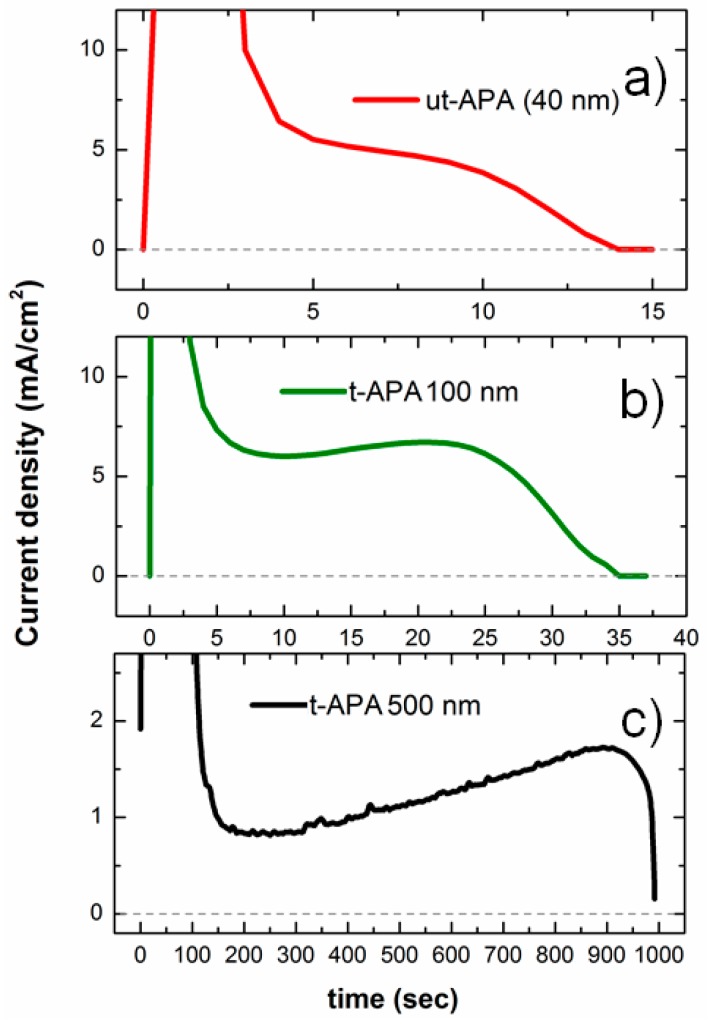
Typical chronoamperometric curves measured during anodization of Al on glass substrates for ultra-thin anodic porous alumina (utAPA, (**a**) thickness 40 nm) and thin anodic porous alumina (tAPA) with different thickness ((**b**) 100 nm and (**c**) 500 nm).

The anodization mechanism of thick Al foils is well-known, and different process stages leading to pore formation have been identified [2]. The time scale of this process is usually of some hours, depending on the thickness of the anodized Al layer, which may reach several tens of micrometers. When performing the anodization on a much thinner Al layer deposited on a substrate (either Si or glass), we are significantly changing the process conditions, which can also give rise to side effects such as modifications of the substrate itself [20]. An easy way to follow the electrochemical process as it is proceeding is to measure the current flowing inside the anodization cell. Representative chronoamperometric curves acquired during the anodization of Al films of increasing thickness on glass are presented in Figure 1. In the following, we will assign the thickness of the starting Al (for thin APA) to the APA resulting from its full anodization. Even though this is not exactly correct, it represents a good approximation, because the Pillig–Bedworth ratio for the anodization of Al is ~1.3 [21], and some thickness may be lost after excess APA etching due to, e.g., overheating following inefficient anodization. Furthermore, this makes it easier to distinguish the APA according to the starting Al. Obviously, for thick APA, which will be called simply APA, not the whole Al foil is completely anodized, which would take several days.

As expected, the shapes of the presented current density profiles in Figure 1 are all similar, and the main difference among them is the time scale, which ranges from a few minutes for 500 nm tAPA (Figure 1c) down to a few seconds in the case of 100 nm and 40 nm tAPA (Figure 1b,c, respectively). It should be noted that the control of anodization of an ultra-thin Al layer is not trivial, since the stages leading to the formation of pores and their growth at equilibrium take place within a limited time, up to a maximum of a few tens of seconds. Still, the process has to be long enough to form the barrier oxide layer and start the competing process of etching/growth, which gives rise to the columnar pores. The shape of the anodization curve is a simple way to verify that the pore formation has taken place. In the case of a thick Al foil, the current density is comparatively high, and stays nearly constant for the whole process until the power supply is turned off by the operator [22]. The thickness of APA grown at the expenses of the (virtually infinitely thick, >200 μm) Al foil is roughly proportional to the anodization time [23]. In the case of tAPA and utAPA (black, green, and red lines in Figure 1, corresponding to the full anodization of 500-nm, 100-nm, and 40-nm-thick Al, respectively), the current, after an initial spike when there is no oxide yet, keeps a roughly constant level until the whole Al film is consumed and converted into APA, a point at which the current goes to zero. As far as utAPA is concerned, even though the process spontaneously stops after about 15 s, the shape of the anodization curve is maintained, suggesting that the pores have indeed formed.

The use of an insulating glass substrate below the Al layer allows for better control of the process, since the anodization is self-terminated when the current drops because of the total consumption of the Al (see Figure 1). Nevertheless, the current does not go down to zero when using a semiconductor substrate. In fact, the use of Si substrate was found to give rise to undesired APA pore bottom reversal due to the growth of Si oxide [20,24]. In addition, it is interesting to point out that, even though the 500-nm (black line in Figure 1) and the 100-nm (green line) tAPA have been fabricated using different acids and voltages (see Materials and Methods), the shape of the chronoamperometric curves is similar, meaning that the pore formation process is the same.

**Figure 2:**
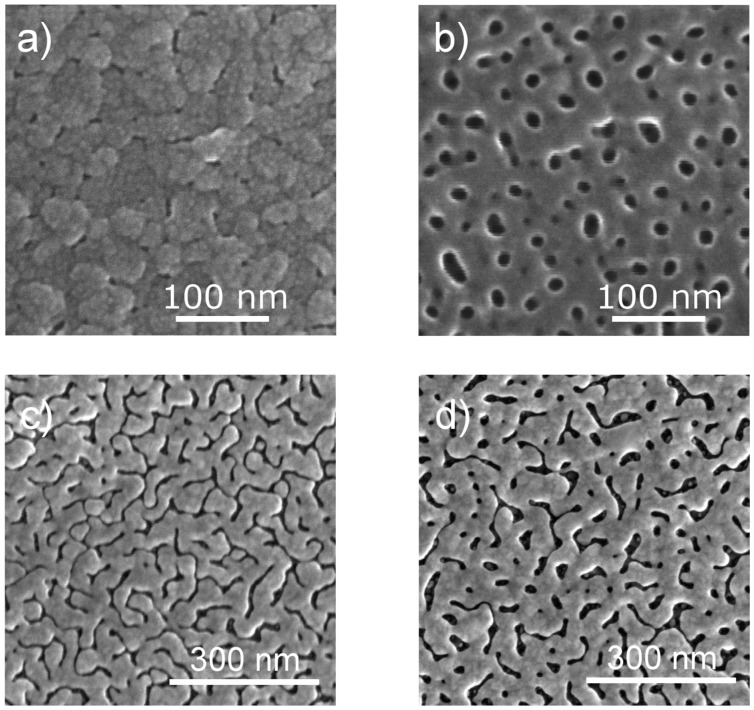
Scanning electron microscopy (SEM) images acquired on utAPA anodized at 20 V in sulfuric acid. Images were taken on the sample (**a**) as-anodized and (**b**) after pore opening; Panels (**c**) and (**d**) show the resulting morphology of the same samples after coating with a 20 and 25-nm-thick gold (Au) layer, respectively.

The anodization curves of utAPA indicate a possible growth of pores within the oxide layer. The morphology of utAPA was therefore investigated via scanning electron microscopy (SEM). Representative images acquired on ultra-thin Al films anodized with different protocols are presented in Figure 2. The image in Figure 2a was acquired on utAPA anodized from a ~30-nm-thick Al layer evaporated on glass using a sulfuric acid solution and a 20-V constant voltage. The corresponding anodization curve is shown in green in Figure 1. Here, the pores are hardly distinguishable, and the grainy morphology of the evaporated Al film is still visible. Therefore, it was not possible to extract the average morphological parameters such as the pore size (d), the pore pitch (D), and the thickness of the wall between two adjacent pores (w), as done in previous studies [10,11]. After the pore widening process (8 min at room temperature), the pores are clearly visible, as can be seen in Figure 2b, even though they are smaller as compared to those obtained on tAPA (similar SEM images on tAPA were presented in [10,11]. The mean pore size is estimated to be 13 nm, and the cell size is ~40 nm. The morphological parameters estimated for thick APA, tAPA, and utAPA are presented in Table 1. It should be noted here that the space for tuning the pore size is limited in the case of utAPA, because the small thickness prevents the performance of a long widening process, which could cause a complete etching of the oxide layer. This limitation could be addressed by depositing an initially thicker Al layer and performing two-step anodization, which nevertheless would complicate the process and thus limit its possible industrial applicability in view of applications. The tunability of the pore size, one of the biggest advantages of APA, is therefore limited for single-step anodization utAPA.

If we review the values reported in Table 1, we can notice that not only the pore size d obtained with utAPA is smaller than the one obtained for tAPA, but also the cell size D is smaller. Therefore, the density of pores is higher in utAPA.

In order to perform SERS measurements, utAPA must be coated with a 20-nm layer of Au (utAPA-Au). As shown in previous works on thin and thick APA [10,11,18], the Au layer, mimicking the topography of the underlying APA, shows a remarkable enhancement of the Raman scattering of a molecule adsorbed on it. Hot spots are in fact generated on the edge of the pore walls in the metallic layer [18]. The Raman signal enhancement achievable in SERS has a strong dependence on the sizes of the pores, their density, and arrangement [10,18].

SEM images acquired on Au-coated utAPA samples are presented in the panels of Figure 2c,d. Especially in the case of the as-anodized sample (Figure 2c), the Au pattern presents features that look more like grooves than pores, showing little resemblance to the respective utAPA lying beneath it (see Figure S1a). The lack of correspondence between gold-coated and “nude” APA was unexpected since it did not occur with tAPA-Au [10,11,18]. A possible explanation of the observed difference in the pattern could be found in the higher surface roughness of utAPA with respect to tAPA. In fact, the shorter anodization is not sufficient to etch all the asperities of the deposited Al grains (see Figure S1b for an image of the starting Al film), and the deposited metal layer probably tends to follow the residual Al roughness more than the pore profiles. The APA pore patterns have been subjected to much image analysis and characterization [25,26,27], which can give some insight in the correspondence between fabrication conditions and the resulting geometry. However, the pattern characterization, as well as the explanation of its origin, is beyond the scope of the present work, which aims to identify reproducible procedures to fabricate the most efficient SERS substrate based on APA-Au nanostructures.

### 2.2. SERS Measurements

**Figure 3:**
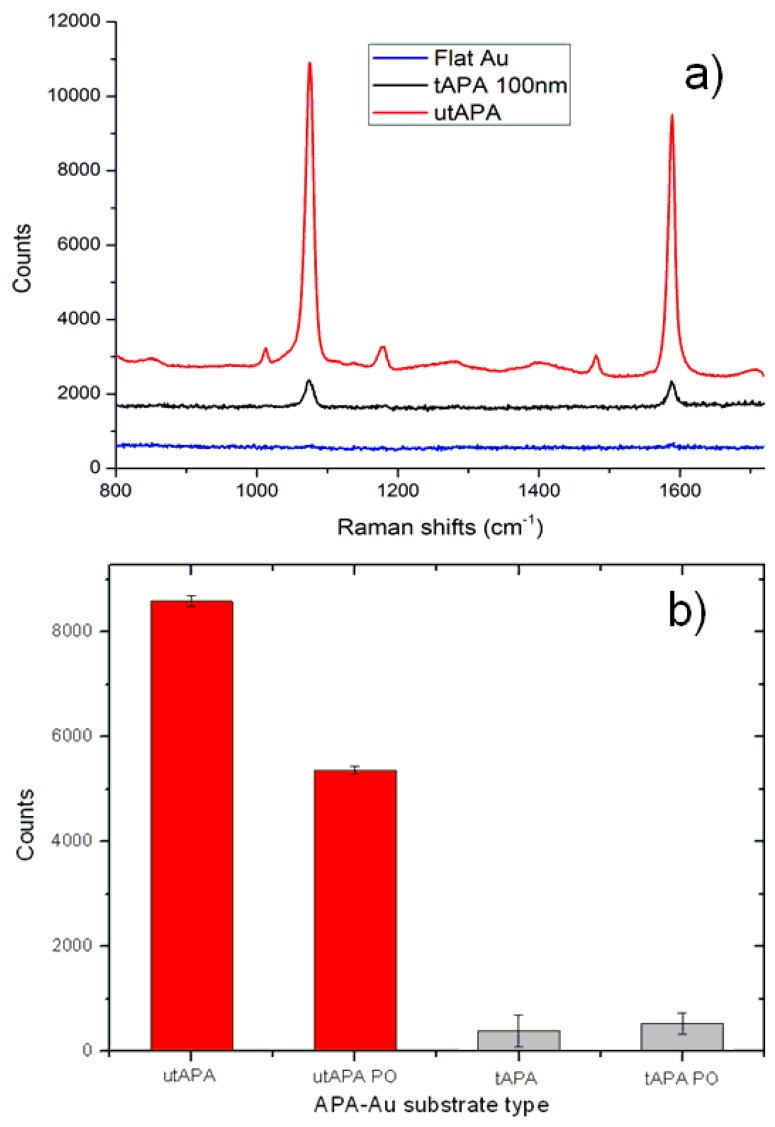
(**a**) Typical Raman spectra of test probe molecule mercaptobenzoic acid (MbA) on utAPA-Au (red) tAPA-Au (black) and Au (blue); (**b**) Peak intensity at 1076 cm^−1^ extracted from the Raman spectra of test probe molecule MbA on different substrates of tAPA and utAPA, before (no suffix) and after pore opening (PO suffix). Bars are mean ± standard deviation (*N* = 8).

Mercaptobenzoic acid (MbA) was used as the target molecule for SERS experiments, since it forms self-assembled monolayers on gold via thiolate derivative, and it has characteristic Raman features. SERS spectra of MbA acquired on utAPA-Au and tAPA-Au together with the Raman spectrum obtained on flat Au are presented in Figure 3a. The mean curves, resulting from averaging spectra on different zones of the samples, show characteristic Raman bands located at 1076 cm^−1^ and 1580 cm^−1^, assigned to aromatic ring vibrations [28].

A very important aspect when developing a SERS substrate is its spatial uniformity. In other words, the signal enhancement must occur over the whole sensor area and should not show significant variations. 2D mapping measurements were performed to check the samples uniformity (see Figure S2), and confirmed that utAPA-Au can provide a SERS effect over a large area.

The peak intensity obtained by fitting the spectra with a Lorentzian function centered at 1076 cm^−1^ are displayed in Figure 3b. The MbA peaks obtained on utAPA-Au are more intense than the ones acquired on tAPA-Au in the same conditions, both before and after pore opening. Since the pore arrangement and shape is different between the two sets of samples, it is not clear whether the better performance of the utAPA is due to the lower oxide thickness or to the different surface morphology. This point will be addressed later when discussing the insights provided by the simulations. In addition, from Figure 3b, it appears that the as-prepared substrate (utAPA-Au) shows better performances in terms of signal enhancement with respect to the pore opened one (utAPA PO-Au), possibly due to the fact that the grooves are thinner, and the separation between the walls is therefore shorter. In fact, since previous theoretical studies on ordered thick APA [18] pointed out that hot spots occur at the edge of the pores, it is plausible that a smaller separation between the walls would cause an increase in the electric field enhancement. However, since the topography of utAPA and tAPA are quite different from the ones previously simulated, we performed simulations on the newly obtained structures in order to estimate where hot spots are located and with what density.

### 2.3. Numerical Simulations

**Figure 4:**
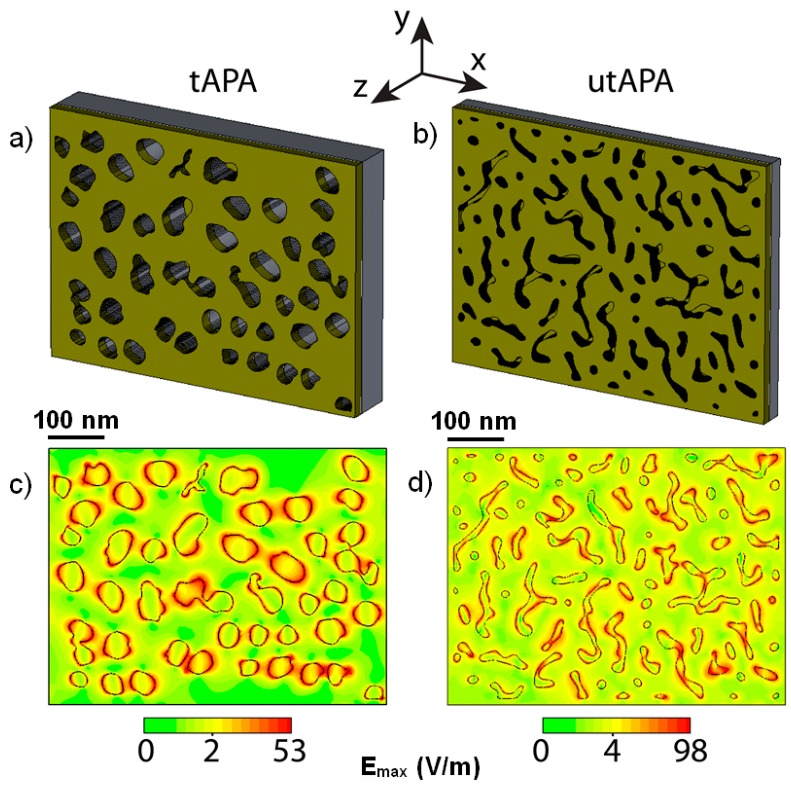
Simulations of the electric field intensities on the APA-Au nanostructures in the cases of the respective maxima, both occurring close above the bottom Au layer inside the pores: (**a**,**c**) tAPA (80-nm APA thickness, 25-nm Au thickness, vertical position from bottom z = 30 nm, E_max_ = 58); (**b**,**d**) utAPA (30-nm APA thickness, 20-nm Au thickness, vertical position from bottom z = 20 nm, E_max_ = 163).

Simulations using CST software, a numerical tool based on the Finite Integration Technique, were performed to evaluate the near field electromagnetic distribution within utAPA-Au and tAPA-Au structures. The model was built considering an Au layer on top of an APA layer with thicknesses of 30 nm (utAPA) and 80 nm (tAPA) and with pores arranged as in both characteristic patterns of tAPA-Au and utAPA-Au. The simulations were not performed on ideal structures; rather, actual representative SEM images with randomly distributed pores were used. The Au thicknesses were slightly different in the two cases, namely 20 nm for utAPA and 25 nm for tAPA, as in the actual samples. In Figure 4a,b, the 3D reconstruction of the simulated tAPA-Au and utAPA-Au structures are presented. It must be noted that, in the simulations, we considered that Au is actually penetrating within the pores; therefore, a 20 or 25-nm-thick layer (for utAPA or tAPA, respectively) was added to the bottom of each pore. The excitation source is a linearly polarized plane wave with a 785-nm wavelength, which impinges on the sample perpendicular to the surface.

Figure 4c,d show the simulated electric field distribution in tAPA-Au and utAPA-Au, respectively, at the depth inside the structure that gives the highest maximum electric field intensity, respectively. In both cases, this occurs close above the bottom Au layer, inside the pores. As for the lateral distribution of the hot spots in the respective pattern, it is clear that the regions where the electric field is higher (red areas), reaching the maximum value E_max_, are closely related to the presence of the pores. As expected [18], the hot spots are located at the edge of pore walls and are randomly distributed within the structure.

**Figure 5:**
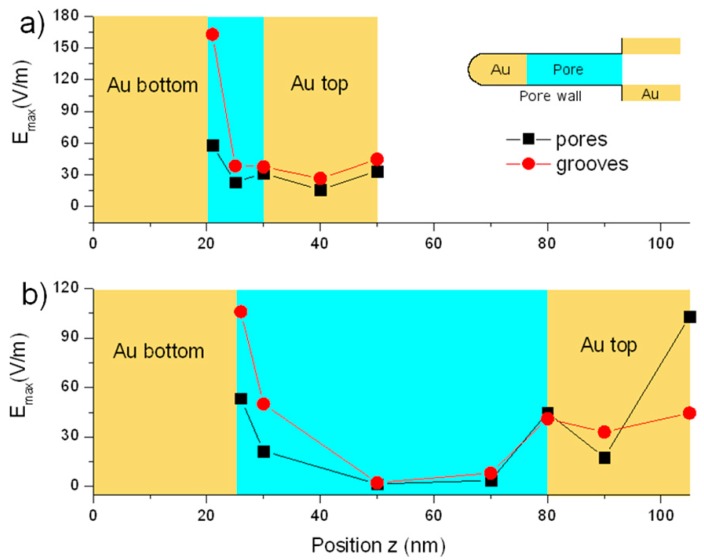
E_max_ values derived from simulations performed at different height *z* within the APA-Au structure. Total thickness is APA thickness + Au coating thickness (25 nm for tAPA and 20 nm for utAPA). (**a**) 30 nm-thick APA; (**b**) 80 nm-thick APA. The coated Au has penetrated the APA and reached the bottom of the pores, as shown in the inset to (**a**). The black curves correspond to a pore arrangement similar to the one of tAPA (pores) while the red ones correspond to the morphology of utAPA (grooves).

The simulations were actually performed at different height values (*z* values) measured from the bottom-most depth within the structure, with the aim of understanding whether the Au layer at the bottom of the pores is playing a role. Figure 5 displays the E_max_ values found for each simulation performed at different *z* in the case of 40 nm utAPA (Figure 5a) and 80 nm tAPA (Figure 5b). The yellow-colored regions represent the Au on either the top or the bottom of the APA pores. Obviously, these conditions (Au on top or on bottom) alternate throughout the structure (see inset of Figure 5a); however, for simplicity, we represented them in Figure 5 as uniform bands. For both thicknesses, we considered either a pore distribution pattern similar to real tAPA, namely pores (black curves), or a surface morphology similar to real utAPA, namely grooves (red curves). The actual penetration of the deposited Au down to the pore bottom was confirmed in both cases of tAPA and utAPA by SEM imaging of sacrificial sample cross sections (see Figure S3).

Therefore, it should be noticed that the simulations cover both real cases (utAPA with 30-nm thickness and groove pattern, and tAPA with 80-nm thickness and pore pattern) and two additional imaginary cases, where one would ideally obtain the same patterns with inverted thickness, namely pores in utAPA (30-nm thickness) and grooves in tAPA (80-nm thickness). While the latter could not be obtained in reality due to fabrication issues, simulations may still help in assessing their potential efficiency, thus giving an indication of their possible advantage. We see from the results that in any case the best condition is the real one obtained with utAPA and grooves.

The real utAPA pattern of grooves seems to work better than the tAPA pattern of pores in all cases but for the z corresponding to the free surface of the Au top for tAPA (rightmost datapoints in Figure 5b). For both patterns (pores and grooves) and APA thicknesses (tAPA and utAPA), the signal inside the APA is at maximum on the Au at the bottom of the pores (leftmost datapoints in Figure 5a,b)—~4× for utAPA and ~10× for tAPA. Thus, in order to take the best advantage of these structures, one should be able to load the SERS ‘probes’ down to the pore bottom.

For the utAPA pattern of the grooves in particular (Figure 5a, rightmost red circle), there is a strong effect of the Au on the pore bottom. Indeed, the electric field (E_max_) increases progressively, moving down deeper into the APA thickness, and becomes highest in the case of utAPA.

In Figure 5, the electric field values for the pore pattern (black squares) are relatively constant for different z positions, as compared to the groove pattern (red circles), for both tAPA and utAPA thicknesses. When the maximum signal for both real cases (tAPA-pores and utAPA-grooves) appears, *i.e.*, on top of the Au at the pore bottom, E_max_ ~ 58 for tAPA and ~163 for utAPA. The ratio of these values is ~2.8× in favor of utAPA. Thus, since the electromagnetic signal intensity scales as the square of the electric field value, the SERS signal for utAPA substrate with grooves would be ~8 times higher than for tAPA (with pore pattern) in the case of the enhancement of only one intensity (incident or scattered), whereas it would be ~62 times higher for the enhancement of both above intensities [29]. Our experimental values of ~20× relative enhancement are intermediate and therefore compatible with a mixed situation as compared to these simulations. Finally, as to the absolute values of experimental enhancement observed, they are 200–250 for tAPA and 4000–5000 for utAPA.

## 3. Materials and Methods

### 3.1. Au-utAPA Substrate Preparation

tAPA and utAPA were obtained by anodizing thin (thickness 100–500 nm) and ultra-thin films (thickness < 40 nm) of Al. The metal was deposited on borofloat glass wafers (SWI, Hsinchu, Taiwan) with a size of 2 in and an optically polished surface, using an electron-beam evaporation (PVD75 evaporator, Kurt J. Lesker Ltd., Hastings, UK) at a base pressure of 10^−6^ Torr and a deposition rate of 0.3 Å/s.

The anodization of utAPA was performed at constant voltage (20 V) in a homemade Teflon cell, in a 1 M sulfuric aqueous solution (bath temperature 15 °C), by contacting the sample from the front via adhesive copper tape, embedding the edges of the substrate, and touching a stainless steel plate on the bottom. Due to the small thickness of the metal film, the anodization was carried out in a single step, yielding a fully oxidized layer in about 10 s. A similar protocol was used to fabricate tAPA, as described in [10,11]. In both cases, the process is self-terminating, because the current goes to zero as soon as the metal is completely oxidized. A first indication that the oxidation has taken place in the whole metal layer is that the sample becomes transparent. In addition to the as-fabricated samples, we also prepared a batch of samples with increased pore sizes (d) and fixed pore pitches (D). The pores were widened by wet etching in a 9 wt % phosphoric acid solution at RT for 8 min. Longer etching times led to the disruption of the ultra-thin oxide.

The samples were coated with either a 20-nm or 25-nm Au film, depending on the experiment, using an Emitech K950X high vacuum turbo system (Quorum Technologies, Lewes, UK). The metal coating was used to perform SERS measurements, as already done in some previous works [10,18] and SEM measurements on the insulating samples.

High-resolution SEM was carried out on utAPA using a JSM-7500F (Jeol, Tokyo, Japan) equipped with a cold field emission gun, collecting secondary electrons. The accelerating voltage of 15 or 5 kV was used, according to the sample surface condition.

The estimation of the morphological parameters of the APA patterns was performed using the grain analysis tool of Igor 6.22 (Wavemetrics, Tigard, OR, USA). All the images (1280 × 960 pixels, 8-bit grayscale intensity) were pre-treated to avoid erroneous assignment of single pixel noise to tiny pores: a low-pass filter with a spatial kernel filter of Gaussian profile and $\sqrt[{2}]{\text{pixel}}$ radius was applied by means of Photoshop CS5 (Adobe Systems Inc., Mountain View, CA, USA). The mean d and D values were obtained by averaging the values from three different SEM images of the same representative sample.

### 3.2. Raman Measurements

Au-tAPA and Au-utAPA substrates were incubated in a 1-mM ethanol solution of 4-mercaptobenzoic acid (MbA, Sigma, Milan, Italy) for 2 h, and then gently rinsed with ethanol and dried under nitrogen flow. SERS investigations were performed using a micro-Raman spectrometer (inVia Raman microscope, Renishaw, Gloucester, UK) and a 785-nm laser line (output power ~100 mW, power on the sample ranging from 0.5 to 50 mW), a grating with 1200 grooves/mm^−1^, and a 50× objective (NA 0.75). Spectra were collected in the 720–1800 cm^−1^ spectral range. In order to verify the uniformity of the samples, 2D Raman mapping was also carried out.

### 3.3. Simulations

The numerical simulations, performed with CST software, are based on the finite integration technique (FIT), first introduced by Thomas Weiland in 1977 [30]. FIT is based on the solution of the Maxwell equations in their integral form. This approach allows for the division of the overall simulation domain in smaller portions (units), with each of them being separately solved. Continuity relations among adjacent units provide the consistency of the general electromagnetic solution. As all numerical solvers, FIT simulations also strongly depend on the chosen mesh. In the present case, we have adopted a tetrahedral mesh, which is especially suitable for curved surfaces. In particular, the convergence analysis approach has been especially stressed due to the strongly irregular morphology of the structure. In this way, the error drops below 5% on the near-field calculations.

## 4. Conclusions

SERS using APA as an efficient substrate, thanks to its large area capabilities and inexpensive fabrication procedure, has been a topic of current interest for several years. In this work, we have presented one step towards even more efficient APA-based SERS substrates based on the fabrication of APA layers, called utAPA, thinner than 100 nm. The work has been supported by modeling and simulations of the electric field appearing on our APA nanostructures when coating them with Au and exciting them with laser light. For these photonic nanostructures, simulations are important to provide a deeper understanding of the enhancement mechanism and to guide us towards progressively better results. In the case of our test analyte, consisting of molecules of MbA, we observed values of Raman signals increased by ~20 times on utAPA, as compared to thicker APA, tAPA. Similar relative enhancement values were confirmed by the simulations and ascribed to the resonant coupling between the Au layer on the top of the APA walls and that penetrated on the bottom of its pores. Such an improvement in signal intensity observed here can help in ultrasensitive applications such as in chemical sensors, biosensors, and bioassays.

## Figures and Tables

**Table 1:** Typical geometric figures of the fabricated utAPA *vs.* tAPA of 100 and 500 nm.

APA Type	Protocol	V (V)	D (nm)	σ (10^10^ cm^−2^)	d (nm)	w (nm)	w/d	w/D
tAPA 500 nm	Phosphoric acid	110	247	0.21	66	181	2.74	0.73
tAPA 500 nm	Phosphoric acid + pore opening	110	234	0.23	157	77	0.49	0.33
tAPA 100 nm	Oxalic acid	40	94	1.44	21	73	3.48	0.78
tAPA 100 nm	Oxalic acid + pore opening	40	103	1.20	82	21	0.26	0.20
utAPA	Sulfuric acid + pore opening	20	41	7.6	13	28	2.15	0.68

## Supplementary Materials

The following are available online at www.mdpi.com/1996-1944/9/6/403/s1. Figure S1: (a) Region of scratched Au overlayer on utAPA, showing the effect of pattern mismatch between the underlying utAPA nanoporous structure and the resulting “groove”-like structure of the coating Au; (b) Image of the typical Al layer from which anodization is started to obtain all the APA with different thicknesses investigated here. The quality is quite consistent (from AFM images, not shown, it turns out that the RMS roughness S_q_ is ~12 nm, for a scan size of 10 μm); Figure S2: Typical Raman map (red area) on a 64 µm × 64 μm (8 μm step) region of a utAPA-Au substrate with MbA adsorbed on it (the bluish area is the optical image of the sample). The map displays the intensity of the MbA Raman peak at 1076 cm^−1^, which is quite uniform on a comparatively large area; Figure S3: Representative SEM cross-sectional image of tAPA overcoated with Au. The Au pads reaching the bottom of the APA pores are clearly visible. This situation is typical for all the considered APA substrates, including those of utAPA. The image has been acquired in “additive mode”; therefore, it contains both morphological (secondary electrons) and compositional (backscattered electrons) information.

## Abbreviations

The following abbreviations are used in this manuscript:

APA	anodic porous alumina
tAPA	thin APA
utAPA	ultra-thin APA
Al	aluminum
Au	gold
Si	silicon
FIT	finite integration technique
SERS	surface-enhanced Raman scattering
