# Supplementary material for: Fabrication of Gold-Coated Ultra-Thin Anodic Porous Alumina Substrates for Augmented SERS
Source: Materials (Basel). 2016 May 24;9(6):403. doi: 10.3390/ma9060403 (PMC5456800; doi:10.3390/ma9060403)
Supplement: Supplementary file 1 [file materials-09-00403-s001.pdf]

Supplementary Materials: Fabrication of Gold-Coated Ultra-Thin Anodic Porous Alumina Substrates for Augmented SERS

Chiara Toccafondi, Remo Proietti Zaccaria, Silvia Dante and Marco Salerno

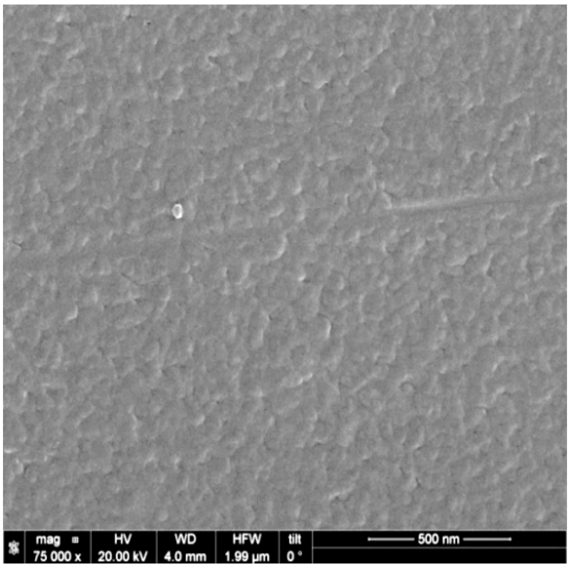

(**a**)

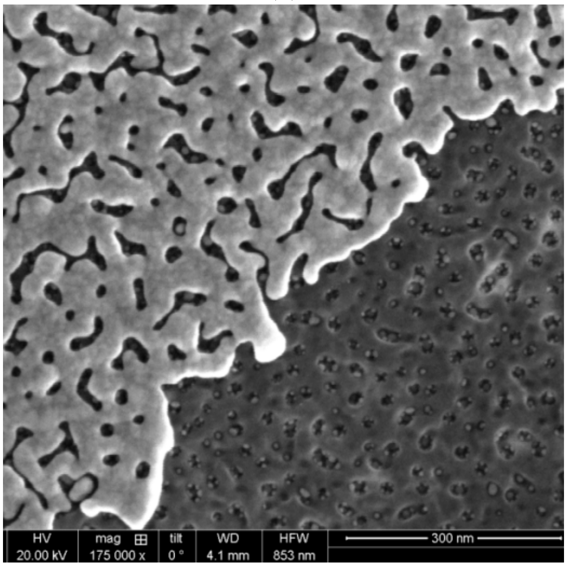

(**b**)

Figure S1. (**a**) Region of scratched Au overlayer on utAPA, showing the effect of pattern mismatch between the underlying utAPA nanoporous structure and the resulting "groove"-like structure of the coating Au; (**b**) Image of the typical Al layer from which anodization is started to obtain all the APA with different thicknesses investigated here. The quality is quite consistent (from AFM images, not shown, it turns out that the RMS roughness S_q is ~12 nm, for a scan size of 10 μm).

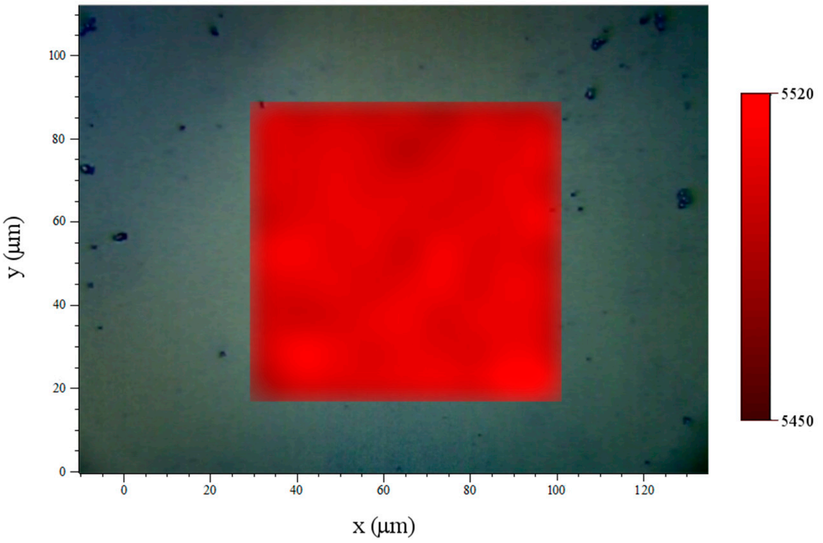

Figure S2. Typical Raman map (red area) on a 64 μm × 64 μm (8 μm step) region of a utAPA-Au substrate with MbA adsorbed on it (the bluish area is the optical image of the sample). The map displays the intensity of the MbA Raman peak at 1076 cm^{-1}, which is quite uniform on a comparatively large area.

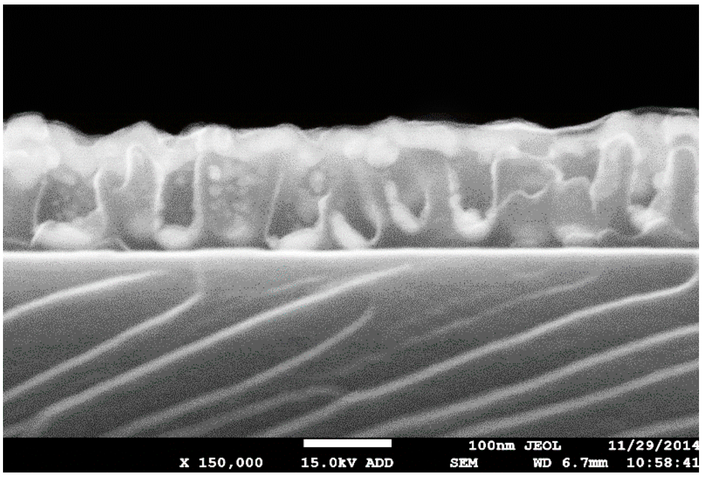

Figure S3. Representative SEM cross-sectional image of tAPA overcoated with Au. The Au pads reaching the bottom of the APA pores are clearly visible. This situation is typical for all the considered APA substrates, including those of utAPA. The image has been acquired in "additive mode"; therefore, it contains both morphological (secondary electrons) and compositional (backscattered electrons) information.
